# The Regulation of Task Performance: A Trans-Disciplinary Review

**DOI:** 10.3389/fpsyg.2015.01862

**Published:** 2016-01-07

**Authors:** Ian Clark, Guillaume Dumas

**Affiliations:** ^1^Nagoya University of Commerce and BusinessNagoya, Japan; ^2^Human Genetics and Cognitive Functions Unit, Department of Neuroscience, Institut PasteurParis, France; ^3^Synapses and Cognition, UMR3571 Genes, Centre National de la Recherche ScientifiqueParis, France; ^4^Human Genetics and Cognitive Functions, Sorbonne Paris Cité, University Paris DiderotParis, France

**Keywords:** meta-cognition, self-regulation, self-efficacy, self-reflection, prefrontal cortex

## Abstract

Definitions of meta-cognition typically have two components: (1) knowledge about one's own cognitive functioning; and, (2) control over one's own cognitive activities. Since Flavell and his colleagues provided the empirical foundation on which to build studies of meta-cognition and the autonoetic (self) knowledge required for effective learning, the intervening years have seen the extensive dissemination of theoretical and empirical research on meta-cognition, which now encompasses a variety of issues and domains including educational psychology and neuroscience. Nevertheless, the psychological and neural underpinnings of meta-cognitive predictions and reflections that determine subsequent regulation of task performance remain ill understood. This article provides an outline of meta-cognition in the science of education with evidence drawn from neuroimaging, psycho-physiological, and psychological literature. We will rigorously explore research that addresses the pivotal role of the prefrontal cortex (PFC) in controlling the meta-cognitive processes that underpin the self-regulated learning (SRL) strategies learners employ to regulate task performance. The article delineates what those strategies are, and how the learning environment can facilitate or frustrate strategy use by influencing learners' self-efficacy.

## Introduction

Meta-cognitive processes were first discussed by psychologists interested in strategies for improving memory and the recall of information (Flavell, 1976, 1979; Flavell and Wellman, 1977). The foundational work of Flavell and his colleagues provided an empirical scaffold upon which to build studies of self-knowledge (Fleming and Dolan, 2012). The intervening years have seen the extensive dissemination of theoretical and empirical research on meta-cognition that now includes a variety of issues and domains including educational psychology. Nevertheless, the psychological and neural underpinnings that determine the accuracy of meta-cognitive reflections, predictions, and the subsequent regulation of task performance are not well understood.

The development of self-regulatory strategies, which permit individuals to exercise control over their own learning so that it leads to successful outcomes is the *sine qua non* of effective schooling. This is because much of the individual variation between learners with regard to task performance can be explained by their capacity of self-control and self-regulation (Hasselhorn and Labuhn, 2011). When learners are “being meta-cognitive” they take charge of their own learning (Hacker et al., 2009), and consciously direct effort toward improving task performance (Harlen, 2006).

Meta-cognition is differentiated from cognition as the latter concerns performing a task (e.g., summing a column of numbers), and the former the regulation of that performance by concurrent processes of monitoring and evaluative reflection on the quality of the performance (Clark, 2012) often referred to as “thinking about thinking.” For example, changing how we perform a task if we feel that a current strategy is not optimal (Garrison, 2014). This two-level framework has extended beyond studies on information recall to encompass the monitoring of perception (Rounis et al., 2010), decision-making (Fleming et al., 2010), sense of agency (Morsella et al., 2009), and learning (Dienes, 2008).

## Toward a trans-disciplinary perspective on educational research

One of the fundamental pillars supporting the link between education and neuroscience is the ability of the brain to learn. This challenges the perspective that the human brain and learning should be viewed in different ways, and further, suggests a neural basis for learning. However, the literature on neuroscience and educational psychology may appear distantly related at best. It is therefore not without controversy that these disciplines are integrated into a framework known as educational neuroscience. A major goal is to bridge the gap between the two fields through a direct dialogue between researchers and educators. These collaborations capitalize on the tensions and synergies between disciplines (as seen taking place at the Centre for Educational Neuroscience in London, UK) in order to create what may be seen as a new *trans*-disciplinary “substance” with implications for classroom practitioners. Educational neuroscience (also called Mind Brain and Education; MBE) is therefore an emerging scientific field that brings together researchers from differing disciplines (just as the writing of this article has done).

Pickering and Howard-Jones (2007) of the UK's Department for Education and Skills (DfES) Innovation Unit surveyed the opinions of teachers and educators from around the world on the applicability of neuroscience to education. The findings indicated that they were generally enthusiastic about the use of neuroscientific findings in the field of education. Indeed, the respondents in the study felt these findings would be more likely to influence their teaching methodology than curriculum content. There is consensus among educational neuroscientists that the link between education and neuroscience is in its infancy. Much work is required in order to apply neuroscientific research findings to education in a practically meaningful way (Goswami, 2006).

This work is well underway. An increasing number of empirical studies and reviews that reveal how the brain facilitates effective task performance have been published (Badre and D'Esposito, 2007; Burgess et al., 2007; Koechlin and Hyafil, 2007; Forbes and Grafman, 2010; Badre et al., 2011; Fleming and Dolan, 2012; Garrison, 2014; Clark and Dumas, 2015). At the turn of the century, in the US, the National Academy of Sciences (2000) advised that “neuroscience has advanced to the point where it is time to think critically about the form in which research information is made available to educators so that it is interpreted appropriately for practice—identifying which research findings are ready for implementation and which are not.” By Goswami (2004), in the UK, Goswami had stated that neuroscience had led to the discovery of “neural markers” that can be used to assess development. These markers are milestones of neural activity against which learners can be compared in order to understand those individual differences in meta-cognitive skills so crucial to successful task performance (Goswami, 2004; Hasselhorn and Labuhn, 2011). Similarly, Willingham (2009) stated, somewhat controversially that, “whether neuroscience can be informative to educational theory and practice is not debatable—it has been.”

## Neural structures recruited during meta-cognition

The notion that the average person uses only approximately 10% of their brain's capacity is a myth. In reality, the performance of complex tasks entails the recruitment of the entire spatial extent of the brain and the vast range of mental facilities that it supports (Kounios et al., 2008; Chein and Schneider, 2012). Recent neuroscience indicates that the full extent of our brain's resources is available at any given moment, however, we access them selectively. The brain recruits specific circuitry in a coordinated way so it may deal with momentary demands (Chein and Schneider, 2012).

**Figure 1:**
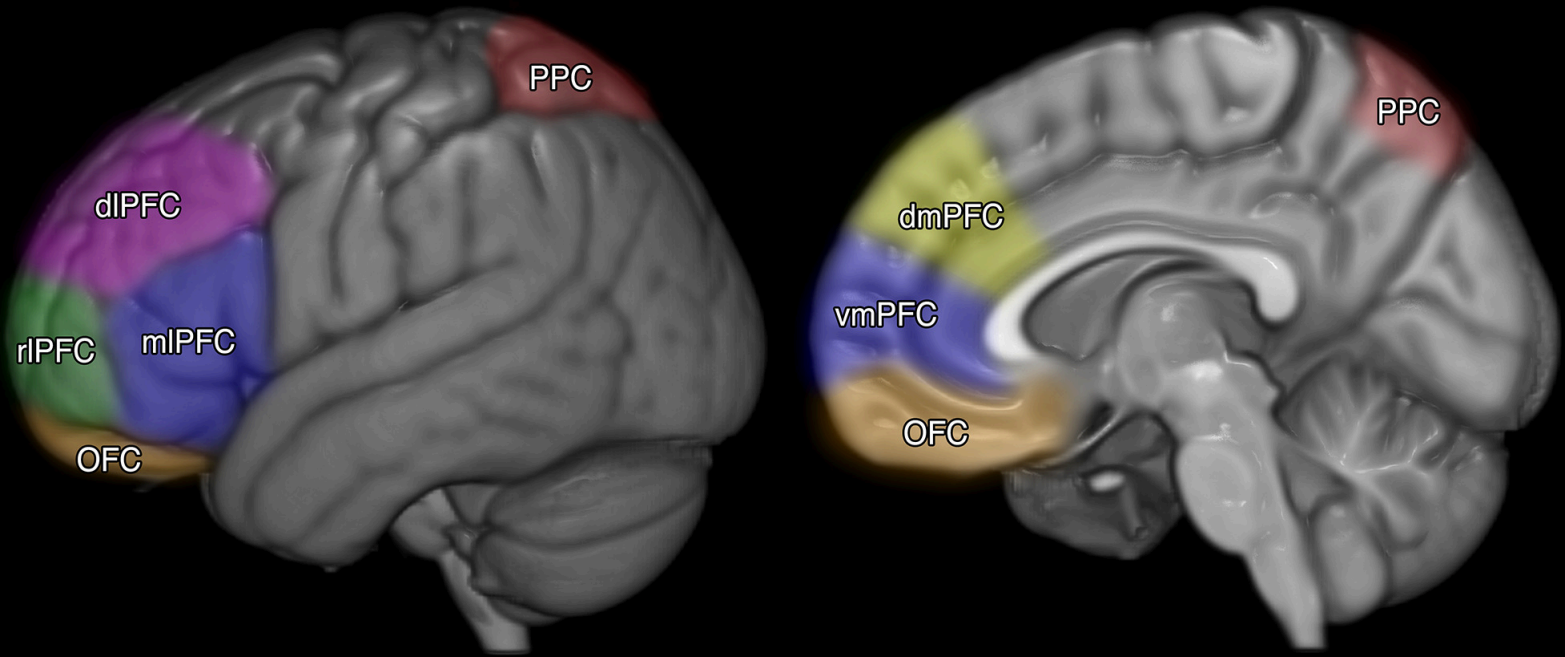
**Key substructures of the prefrontal cortex (PFC) and posterior parietal cortex (PPC)**. PFC to PPC coordination has been associated with many high-level cognitive functions, especially working memory, theory of mind, and meta-cognition.

The prefrontal cortex (PFC; See Figure 1) has been isolated as the core brain region for meta-cognition (Middlebrooks and Sommer, 2012; for review see Fleming and Dolan, 2012). The proposition that meta-cognition is under neural control is supported by a burgeoning amount of neuropsychological literature (e.g., Fleming and Dolan, 2012; Garrison, 2014). Neuroscientific studies have suggested that a defining function of the anterior PFC may be meta-cognitive awareness, or the process of reflection upon one's own mental contents (Nelson and Narens, 1990; Stuss, 2011; Fleming and Dolan, 2012; Garrison, 2014). Evidence from neuroimaging indicates that the PFC operates in concordance with substrates (coordinating but anatomically unconnected areas of the brain) located in the posterior region of the brain (see Figure 1), specifically the posterior parietal cortex (PPC; Ikkai and Curtis, 2011). In developing a meta-cognitive model for mathematical problems solving Anderson et al. (2011) found activity in the PFC and PPC, noting that the PFC was recruited for the monitoring and control over more complex tasks such as geometry and calculus.

The role of the anterior PFC in meta-cognitive judgements of task performance is now “well established” as “pivotal” (Garrison, 2014) by evidence from studies applying convergent methodologies (Del Cul et al., 2009; Fleming et al., 2010, 2012; Yokoyama et al., 2010; Lau and Rosenthal, 2011; McCurdy et al., 2013). Of particular relevance to understanding how the PFC facilitates the conscious control over their learning (see Table 1) are empirical studies that investigate: (a) abstract reasoning (Bunge et al., 2005; Badre and D'Esposito, 2007); (b) the monitoring of internal states (Christoff and Gabrieli, 2000); (c) higher-order aspects of decision-making (Koechlin and Hyafil, 2007; Badre et al., 2011); (d) attentional control (Burgess et al., 2007); (e) moral judgment (Forbes and Grafman, 2010); and even interaction between those aspects (Walton et al., 2004).

**Table 1:** **The characteristics of self-regulated learners and the meta-cognitive strategies they use**.

**Characteristic**	**Strategy**
***These are students who:***	
Self-evaluate	Assessing quality or progress
Keep records and monitor learning	Taking discussion notes/a list of errors
Seek help from adults	Seeking social help from teacher or parents
Self-verbalize	Generating overt/covert prompts to guide learning
Create new learning strategies	Using evidence to adapt and improve learning
Set goals and plan learning progression	Setting and prioritizing goals and sub-goals
Structure the learning environment	Choosing conditions, which make learning easier
Manage time	Regulating progress to realize timely outcomes
Engage in peer learning	Seeking social assistance from peers
Use non-classroom resources	Seeking information, e.g., libraries, Internet
Are persistent and complete tasks	Maintaining activity despite difficulty or distraction
Use self-consequences	Giving self-reward or sanctions based on outcomes
Memorize and rehearse information	Using strategies designed to improve recall
Are self-aware	Being non-judgmentally aware of own shortcomings

The PFC can be divided into a number of sub-structures (see Figure 1). Fleming and Dolan (2012) found how different areas of the PFC are recruited for prospective (predicting) and retrospective (reflecting on) judgment making. When making prospective judgements participants were observed to recruit the ventromedial pre-frontal cortex (vmPFC) due to its apparent role in imagining the future (Sharot et al., 2007). In the case of retrospection (the essential self-regulatory process of meta-cognitive reflection) on past task performances the anterior and dorsolateral PFC are recruited. Schnyer et al. (2004) provided further results supporting vmPFC as a neural basis for self-knowledge. In their study they found that damage to the vmPFC decreased the capacity to make accurate judgements about future performance while leaving the level of self-efficacy unaffected. Moreover, Schmitz et al. (2006) found that individuals with defects or injury in the PFC exhibited deficits in autonoetic knowledge. Thus, the healthy development and functioning of the PFC is essential for accurate self-knowledge, evaluation and reflection (Kruger and Dunning, 1999; Schnyer et al., 2004; Fleming et al., 2014).

## The role of the neural micro-circuit in mediating meta-cognition

**Figure 2:**
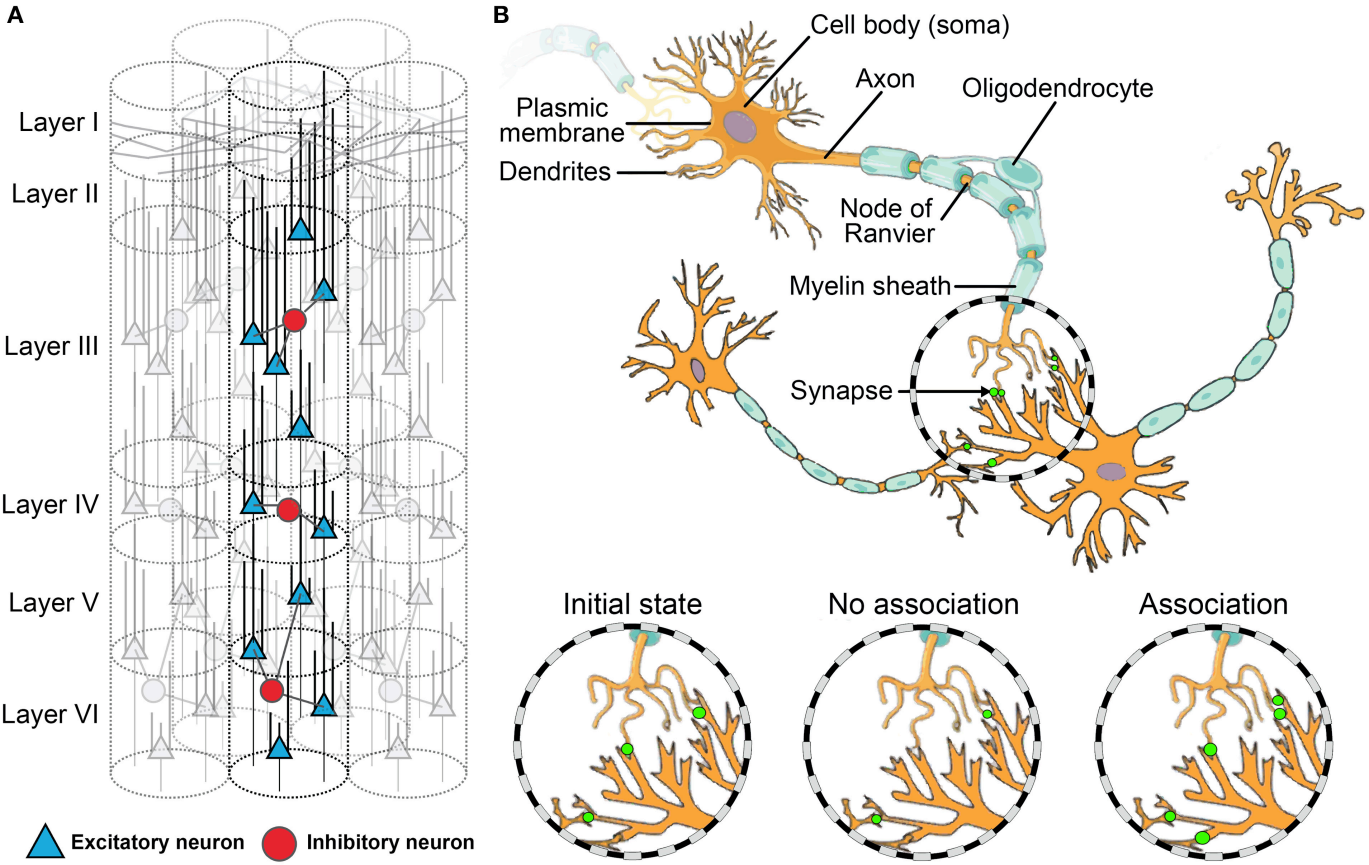
**Neural connectivity and plasticity at the meso- and micro-scale levels. (A)** A cortical macro-column composed of multiple mini-columns. Those ensembles of 80–100 cells across cortical layers form the basic micro-circuit of the brain at the intermediate level between the anatomic and synaptic levels. **(B)** A small network of excitatory neurons (synapses in green). The figure illustrates synaptic plasticity. At the initial state, there are already some synapses. After learning, those existing synapses decrease in size or disappear if there is no association between pre- and post-synaptic neural activity; conversely, if there is an association, the existing synapses increase in size and additional synapses can even appear.

The neural basis for meta-cognition may be represented diagrammatically as three levels: (a) the macroscopic or network level (see Figure 1); (b) the mesoscopic or microcircuit level (Figure 2A); and (c) the microscopic or single-cell level (Figure 2B). Like the rest of the cortex, the PFC is throughout composed of layers of microcircuits (see Figure 2A) which are organized in macro-columns each containing hundreds of mini-columns (Rinkus, 2010). These cortical columns are the most fundamental microcircuits in the brain (Mountcastle, 1997; Horton and Adams, 2005). Each mini-column, (Figure 2A) is composed of hundreds of neurons—pyramidal neurons, which are excitatory, and GABAergic interneuron, which are inhibitory. Those neurons release different types of neurotransmitters such as glutamate for excitatory neurons and GABA for inhibitory interneurons. Moreover, they have different connectivity profiles Goswami: excitatory neurons project both locally and globally (i.e., to other brain areas), while inhibitory interneurons only project locally. Together, they form small networks able to learn patterns depending on the incoming signals from other brain areas, and thus to generate output signals depending on those patterns. Experimental studies have revealed that the PFC activity is highly connected to the rest of the brain, and its activity is thus highly dependent on both high-level contextual information (e.g., rules, strategies) and incoming sensory inputs (e.g., task). Its place as the top hub of the brain can explain the key role that the PFC plays in meta-cognition. The association between those various sets of information in the PFC are supported by changes in these microcircuits (Opris and Casanova, 2014).

Seminal studies have demonstrated how the interplay of global and local dynamics shapes connections between neurons through synaptic plasticity (Changeux et al., 1973). The PFC does not make exception to this phenomenon (Opris and Casanova, 2014). While synaptic pruning and plasticity traditionally underlie associative learning (where ideas and experiences reinforce one another and enhance the learning process; See Figure 2B), the additional level of complexity provided by microcircuits seems to play a role in reversal learning (Deco and Rolls, 2004), cognitive control (Bullock et al., 2009) and flexible contextual planning of action (Constantinidis et al., 2002). These are all key aspects of meta-cognitive learning.

## The development of the PFC across time

Foundational studies (e.g., Flavell, 1979) by developmental psychologists found that self-evaluation and control improved during the course of early childhood. Recent studies of neuroanatomical changes have revealed that the PFC continues to develop throughout childhood and adolescence (Dumontheil et al., 2008). Magnetic resonance imaging (MRI) research has revealed that the PFC changes a great deal during adolescence, as the brain's myelin matures and connects all regions of the brain together. An anatomical feature of particular significance is myelin (see Figure 2B). Myelin is a fatty substance which coats the white matter in the human brain and improves signal transmission to the gray matter so the neurons communicate more quickly. The prolonged development of the PFC is often advanced as the reason for otherwise intelligent and sensible adolescents engaging in high-risk or excessive behaviors even when they understand the potential dangers. Fecteau et al. (2007) demonstrated the influence of the PFC over risk-taking. They diminished risk-taking behavior in healthy participants (*n* = 36) by modulating neural activity in that region.

White matter (60% of brain volume) is therefore made up of nerve cells full of myelin. It works in conjunction with gray matter (only 40% of brain volume, but uses more than 90% of total oxygen), which processes signals originating in the sensory organs and other areas of gray matter.

Increased white matter volume has been observed in young adults (mean age = 27) compared to children (mean age = 10) (Klingberg et al., 1999) demonstrating that maturation of the PFC continues into the second decade of life. Studies find the opposite case for gray matter inside the PFC. Sowell et al. (2004) scanned children (*n* = 45) 2 years apart between ages 5–11. The gray matter decreased significantly over the 2-year period. Similarly, Konrad et al. (2005) observed a reduction in gray matter during adolescence with a smaller gray matter volume in adults (20–34 years). The density of gray matter is associated with intelligence and unique skills (Hogan et al., 2011). Nevertheless, an efficient cognitive functioning relies also on synaptic pruning—a decrease in the number of synapses after adolescence caused by fruitful interactions with the external environment and especially through learning (Craik and Bialystok, 2006). Skilled individuals tend to have high levels of gray matter in the parts of their brains, which correlate to the performance of that particular task. A finding of particular significance regarding gray and white matter volumes is that meta-cognitive ability is correlated with gray matter volume in the anterior PFC, a region that shows marked evolutionary development in humans (Teffer and Semendeferi, 2012) Moreover, inter-individual variation in introspective ability is also correlated with white-matter micro-structure connected with this area of the PFC (Fleming et al., 2010). These findings point to the PFC as a focal neuroanatomical substrate for meta-cognitive processes essential for “good learning.”

## Accurate meta-cognitive judgment

It is the quality of introspection that determines the accuracy of our judgements regarding task performance. In Kruger and Dunning (1999) conducted studies across multiple populations designed to reveal the accuracy of autonoetic knowledge. In tasks including logical reasoning and understanding English grammar the participants were consistently inaccurate about the quality of their own performance. Specifically, they assessed themselves to have scored in the 62nd percentile, yet they had actually scored in the 12th percentile. This is known as the Dunning-Kruger Effect, or what Pronin (2007) terms the “introspection illusion.” McCaig et al. (2011) proved a link between introspection and the PFC with real-time neurofeedback in fMRI. They demonstrated that the participants in their study learned to regulate the rostro-lateral PFC (rlPFC) by using meta-cognitive awareness strategies. It was found that individuals can achieve improved regulation over the level of neural activity in their rlPFC by turning attention toward (or away) from their own thoughts by noticing the nature of any thoughts that appear, such as “planning,” “rehearsal,” and “judging.”

Neuroscientific studies often attempt to measure meta-cognition by asking participants to make judgements before (prospective) and after (retrospective) task performance. Prospective “feelings of knowing” (FOK) are studied by asking participants general knowledge questions, and where they do not know the answer then asking them if they believe that they will be able to select it from a multiple choice set. Another prospective measure of judgment is “judgment of learning” (JOL). This elicits a belief on how much has been learned and will be recalled later. This personal belief that one's efforts will produce successful outcomes equates to self-efficacy. Participants in neuroscientific studies are also required to give retrospective reports on their confidence in their prior judgements regarding task performance. In healthy individuals meta-cognitive judgements are usually predictive of subsequent task performance (Schwartz and Metcalfe, 1996). However, where damage has occurred to the PFC the accuracy of prospective judgment is diminished, yet self-efficacy remains the same (Schnyer et al., 2004). This indicates both a neural basis for accurate levels of self-efficacy and the influence of self-efficacy over the quality of subsequent task performance for healthy learners.

## Teaching strategies for the regulation of task performance

As Garvert et al. (2015) remind in their study of the PFC, “learning induces plasticity in neural networks” (p. 1). By Goswami (2004), neuroplasticity was recognized to be “a fundamental and critical mechanism of neuronal function, which allows the brain to receive information and make the appropriate adaptive responses to subsequent related stimuli” (Duman, 2004, p. 157). Plasticity, or neuroplasticity, describes how experiences reorganize neural pathways in the brain. Long lasting functional changes in the brain occur when we learn new things or memorize new information. According to Nelson (1999) there are 3 mechanisms by which experience causes changes in the brain. The first is an anatomical change, which alters the capacity of existing synapses to modify activity by sprouting new axons, creating or pruning synapses, or by expanding the dendritic surface (see Figure 2B). A second is a neurochemical change, which causes existing synapses to modify activity by improving the synthesis and transmission of signals. Finally, changes in metabolic activity in the brain in response to experience (e.g., the use of oxygen and other nutrients).

All these transformational processes have been widely studied in both human and animal models. The later studies have especially shown how stress and aging alter PFC plasticity (Bloss et al., 2010). Although animal models do not reach the complexity of human meta-cognition experiments, there is some evidence that some animals (dolphins, pigeons, rats, monkeys and apes) do have the meta-cognitive potential to reflect on their cognitive states (Smith, 2009). For humans, however, the plasticity to learn the rules of strategies that facilitate control over cognition also permits leadership in collective settings (agency, as defined by Bandura). This evolving situation requires learners to become active agents who control and shape thinking and learning.

## The development of agency and control

“To be an agent is to intentionally make things happen by one's action…The core feature of agency enables people to play a part in their self development, adaptation, and self-renewal with changing times” (Bandura, 2001, p. 2). Times are indeed changing, and curricula which drive didactic forms of instruction where students are mere passive recipients “do not function in the rapidly changing technological and globalized world of today where it is not possible to establish which type of knowledge is needed in the next 5 or 10 years let alone a lifetime” (Hoskins and Fredriksson, 2008, p. 11).

For learners to be agents they must first believe that they have a mind capable of meta-cognitive control over the performance of a particular cognitive task. For example, children do not try to retrieve events or names before they understand they have a mind able to remember (Carruthers, 2009). Further, when given new information, 3-year old children claim to have always known it (Sodian et al., 2006) and do not know the difference between secure knowledge and making a guess (Gopnik and Astington, 1988). As children develop beyond infancy, they continue to display sub-optimal autonoetic awareness, reporting an over optimism about their capabilities and only rarely reflecting on task performance. As the brain matures into a more “distributed” network (Fair et al., 2009), children as young as 8 begin to accurately self-assess their knowledge (Hasselhorn and Labuhn, 2011). The age range of 7–9 was also found to be a significant developmental milestone in lower order information processing (recall) and higher order meta-cognition (restructuring) in neuroscientific studies by Fair et al. (2009) and Supekar et al. (2010).

As children grow they experience more of the social world by verbally interacting with others. With the emergence of language, they can then explicitly represent their role in a social group and begin to experience a sense of agency. Children also begin to realize that others may be wrong about the world, and use their nascent meta-cognitive skills to monitor and evaluate the accuracy of their own thinking and exercise control over their environment (Frith, 2012). Perhaps inevitably, the development of accurate self- knowledge is accompanied by a decrease in the pleasure derived from learning in learning environments that don't value all student responses, including misconceptions (Clark, 2014). This indicates that inaccurate self-awareness and self-efficacy protect achievement motivation in the early stages of neural development (Hasselhorn and Labuhn, 2011).

As neural structures mature, increasingly complex verbal interactions with others help learners to overcome the lack of direct access to their underlying cognitive processes, thus making their thinking transparent to themselves and also to others. However, it is worth noting, in the context of agency, that not all verbal interaction supports agency. Bandura (1997) emphasizes that teachers should, as the starting point, de-emphasize social comparison and de-personalize feedback because “construal of low attainments as indicants of inherent personal deficiencies erodes a sense of efficacy” (p. 118). The erosion of self-efficacy pushes students into a spiraling pattern of disaffection, which diminishes students' potential to exercise control over task performance.

## Shaping minds and strategy acquisition

It is a fact that most young learners experience a challenging array of distractions and pressures both inside and outside school that often intensify as they travel along the educational pipeline toward high school (see, Burrus and Roberts, 2012). The strategy of sustaining effort in the face of distraction is itself a key self-regulated learning (SRL) strategy (Zimmerman and Pons, 1986; Clark, 2012). Indeed, where learners are unable to exercise control over environmental distractions it follows that other SRL strategies, while theoretically available, may not be accessible for use. Learners may therefore benefit from interventions designed to strengthen SRL strategy acquisition and use. One such program is the “Self-regulation Empowerment Program” (SERP) devised by Cleary and Zimmerman (2004). SERP is an application of socio-cognitive theory, combining diagnostics and training in SRL. The effectiveness of such applications may be assessed by neuroimaging techniques.

The neural plasticity of the PFC implies that programs such as SERP can be implemented in order to train learners how to monitor and reflect on the relationship between strategy selection and task performance. There is a progressive bi-directional relationship between plasticity and the adoption of SRL strategies. As learning induces plasticity, any program designed to support SRL would be highly effective because such learning promotes plasticity that then promotes further strategic mastery. Lodico (1983) found that children who were taught to monitor strategy use chose more effective strategies, and understood that their selection would improve task performance. Evidence from foundational studies in neuropsychology (e.g., Lodico, 1983) and modern neuroscience (e.g., Garvert et al., 2015) indicate that learners may be trained to acquire and use new and potentially powerful self-regulatory learning strategies. If students are to become self-regulated learners, they must first adopt a “growth mind-set” as seen in the work of Dweck (1986, 2006) on fixed vs. growth mind-sets. Dweck finds that individuals who believe that their minds are indeed plastic (growth) are more competent and effective learners than those who believe that they cannot improve with practice (fixed). The question then becomes one of exactly how can learners be given the power to oversee and steer their own learning?

Effective schools encourage a growth mind-set by devising supportive learning environments (Clark, 2014) and delivering curricula that promote learning autonomy (Clark, 2015). Staff members model and scaffold “good learning” by providing written and particularly verbal feedback which explains *how* task performance may be improved, thus scaffolding the learners' control over the re-drafting and self-correction of learning-work (as seen in the work of Black and Wiliam on formative assessment). This kind of feedback is known as “formative feedback” (Black and Wiliam, 2009). The objective of formative feedback is the deep involvement of students in meta-cognitive strategies such as personal goal-planning, monitoring, and reflection, which support SRL by giving learners “the power to oversee and steer one's own learning so that one can become a more committed, responsible and effective learner” (Black and Jones, 2006, p. 8).

## Formative feedback

The essential ingredient of formative feedback is to assist learners to improve task performance by “scaffolding” (Wood et al., 1976) the learning experience by helping them to understand how to improve the quality of their work. Nicol and MacFarlane-Dick (2006) argue that formative feedback should be used to empower students as self-regulated learners. Further, they contend that because formative feedback strategies enhance self-regulation all assessments should be restructured as formative assessments (Sadler, 1989; Nicol and MacFarlane-Dick, 2006). Butler and Winne (1995) underscore the centrality of feedback in regulating learning progression, “for all self-regulated activities, feedback is an inherent catalyst,” (p. 246).

It is worthy of note that not everything that teachers believe to be feedback is in fact formative. For example, Hattie and Timperley (2007) derived effect sizes for formative and non-formative kinds of feedback. They obtain high effect sizes when students are given “formative feedback”; that is, feedback on how to perform a task more effectively, and far lower effect sizes when students are given praise, reward, or punishment. Simply telling a student to “work harder” or “recalculate your answer” does not possess the qualities of formative feedback or promote SRL because it does not strategically scaffold learning by informing the student how or why they need to do this.

Feedback becomes formative when the evidence of learning is used to adapt teaching to meet student needs (Sadler, 1989; Black and Wiliam, 1998). More specifically, students are provided with instruction or thoughtful questioning which scaffolds further inquiry and deepens meta-cognitive processes like reflection. This instructional approach closes the gap between their current level of understanding and the desired learning goal (Vygotsky, 1978). This interactive and mutual process of continual readjustment causes learning to progress at a rate which is sufficient to motivate students to self-regulate the effort required to progress further (Butler and Winne, 1995). This kind of feedback informs students of their current status and how to improve, boosting self-efficacy and task performance. This is so even after students experience typically demotivating initial difficulty performing a task (Assessment Reform Group, ARG; Schraw and Moshman, 1995).

## SRL strategies

In summary, SRL is “an active, constructive process whereby learners set goals for their learning and then attempt to monitor, regulate, and control their cognition,” (Pintrich and Zusho, 2002, p. 250). Self-regulated students are meta-cognitively, socially, motivationally, and behaviorally active in problem solving processes. Students who have been exposed to teaching methods, which reconstruct their identities as self-regulators typically, deploy meta-cognitive strategies in order to exercise control over their work. Zimmerman and Pons (1986) conducted an important foundational study on female and male 10th grade students from a high achievement track (*n* = 40) and from other (lower) achievement tracks (*n* = 40). The researchers created 13 categories of SRL by which students monitored, regulated, and controlled task performance (see Table 1).

The students' membership in their respective achievement group was predicted with 93% accuracy, indicating that membership was determined by the extent to which they exercised accurate and effective control over their learning. Zimmerman and Pons (1986) conclude that “theoretical conceptions of students as initiators, planners, and observers of their own instructional experiences have empirical and practical merit” (p. 626).

## Meta-cognition, self-efficacy, and self-regulation

As already noted, the PFC continues to mature into adulthood. Accordingly, both meta-cognition and SRL are late developing higher-order competencies that are particularly useful when explaining individual differences between young learners (Hasselhorn and Labuhn, 2011). Hasselhorn and Labuhn (2011) explain the inter-individual differences in meta-cognitive competence arise from three influential variables: (1) social influence; (2) the extent and intensity of individual activity; and, (3) the maturity of neural networks. In healthy brains the PFC matures throughout adolescence. This explains why many adolescents demonstrate a sub-optimal grasp of potentially powerful SRL strategy use, which becomes more developed as they approach early adulthood.

The effective use of SRL strategies is not only central to academic achievement; autonoetic knowledge and control over thinking and learning is also essential to meet the demands of a rapidly developing society. Zimmerman and Moylan (2009) define SRL as a sequence of planning, performance and reflection. The planning phase entails task analysis and sources of motivation, especially self-efficacy. It is the level of self-belief and confidence, which determines the difficulty of the learning goals that learners pursue (Bandura, 1997). Implicit to the performance phase is monitoring via processes of self-control and self-observation. Reflection is of particular importance. The essence of meta-cognition is reflection on the individual learning process by comparing the learning outcome with a goal or standard followed by the performance of strategic activities (Hasselhorn and Labuhn, 2011). Reflection creates a pathway toward the use of SRL strategies, and links meta-cognition with learning achievement (as seen in the work of Barry Zimmerman and colleagues).

A theory of particular importance to the development of meta-cognition and the competent regulation of learning is Bandura, 1986 Social Cognitive Theory (SCT), which emphasizes meta-cognition and self-efficacy as fundamental to the development of SRL. Coutinho (2008) found that the relationship between meta-cognition and the regulation of task performance was fully mediated by motivation (self-efficacy). Similarly, Cera et al. (2013) found that meta-cognition was associated with a sense of self-efficacy and that self-regulatory learners possess more advanced meta-cognitive skills and have a high sense of self-efficacy (see Table 2 to see the effect of meta-cognitive strategies on task performance).

**Table 2:** **Summary of intervention-type and effect-size (Dignath et al., 2008)**.

**Intervention**	**Effect-size (*d*)**
Any type of SRL training (i.e., meta-cognitive, cognitive, and motivational)	0.73
Combining meta-cognitive and self-efficacy strategies training (all training)	0.97
Combining meta-cognitive and cognitive strategies (e.g., elaborating by explaining why each fact is true)	0.81
Meta-cognitive strategies only (all strategies)	0.54
Meta-cognitive strategy training in planning and monitoring	1.50
Meta-cognitive strategy training in planning and evaluation	1.46
Training in meta-cognitive reflection (i.e., knowledge about the value of strategies)	0.95
Cognitive strategies training (all strategies)	0.58
Cognitive strategy training in elaboration	1.19
Cognitive strategy training in elaboration, organization, problem solving	0.94
Cognitive strategy training in problem solving	0.72

These empirical findings indicate a need for training and practice in meta-cognitive strategies particularly when combined with motivational and cognitive training, which improves task performance (*d* = 1.50 and 1.46 respectively) via the more active recruitment of the PFC (McCaig et al., 2011). An emphasis on self-efficacy strengthens subsequent meta-cognition. First the existence of, and then the intensity of self-efficacy are central to self-regulation because children need to believe that their minds are capable of successfully performing meta-cognitive processes before they will make even fledgling attempts to regulate their own learning (Carruthers, 2009).

Hasselhorn and Labuhn (2011) note that an increasing body of empirical research has proved the strong link between the capacity to self-regulate learning, and key variables of personal development, such as self-efficacy. From a socio-cognitive perspective, as seen in Bandura's SCT, self-efficacy plays a key role. It is widely held that the level of self-efficacy influences the use of self-regulatory processes such as planning, goal-setting, accurate strategy selection and self-evaluation; all of which influence academic performance. For example, Zimmerman and Kitsantas (2005) found that the level of self-efficacy among adolescent girls (*n* = 179) predicted their use of SRL strategies (e.g., organizing, memorization and rehearsal, monitoring).

Black and Wiliam (2009) in their work on formative feedback emphasize that effective classroom dialogue “is concerned with the creation of, and capitalization upon, “moments of contingency” in instruction for the purpose of the regulation of learning processes” (p. 10). The development of a “moment” into a genuine opportunity for learning is dependent on the meta-cognitive awareness of students' and the accurate self-belief that their efforts will result in success (self-efficacy). A concept of particular relevance is “reflection-in-action”; that blend of monitoring and reflection which together permit the reshaping of that being worked on while working on it (Schön, 1987).

There are also inter-individual differences in the sensitivity of self-reactive judgment to external feedback. This then impacts the level of effort they invest in a completing a task. It can be seen in Table 1 that being non-judgmentally aware of one's own shortcomings is a characteristic of successful learners. Similarly, Bandura and Cervone (1983) found that the increase in effortful behavior following feedback on substandard performance is greater for individuals who have high self-efficacy than in their non-self-regulated counterparts. It follows from this finding that if instructional feedback is to contribute jointly to self-regulation and achievement, teachers should carefully plan for how they will use questioning and feedback which supports the self-efficacy of the student, i.e., scaffolds their learning so it is the students who believe that they are leading, or at least participating in, the discussion or solving the problem as an active agent in their own learning. If this is done regularly, the learner will generate internal feedback, which makes them more self-efficacious and self-regulated (Clark, 2012).

## Environmental stress and the self-regulation of learning

Students' brains react strongly to the school environment; they indeed report emotions that range from apathy to anger (Gilman and Anderman, 2006), and which in many cases may be perfectly appropriate responses to their environment. Therefore healthy neurological functioning needs to take place in a social and learning environment in which students feel supported psychologically because negative emotional states “can lower efficacy beliefs; the lowered beliefs, in turn, weaken motivation and spawn poor performance” (Bandura, 1997, p. 113). Bandura (1997) connects a high sense of self-efficacy with a forward-looking outlook and the tendency to set personal goals. As an individual's perception of their self-efficacy becomes more definite the goals become higher and are more persistently pursued as realizable opportunities. Environmental stress plays a key role in PFC development and functioning; as Blair (2010) notes, environmental stress may “impede the development of reflective and goal-directed self-regulation of behavior such as that needed for success in schools.”

A number of studies on interactive learning environments emphasize the need for supportive environments that reduce psychological stress and distress (Bandura, 1997; Nicol and MacFarlane-Dick, 2006; Clark, 2012). The negative impact of stressful environments is elucidated upon by recent neuroscientific studies. For example, Holmes and Wellman (2009) found that exposure to even brief periods of intense stress is sufficient to cause significant structural remodeling of the neurons within the PFC, impairing cell communication and causing a significant degree of dysfunction to the regulation and control of cognition. These findings confirmed earlier studies by Weinstock (2001) and Meaney and Szyf (2005). Chronic early stress alters neural functioning and connectivity among various structures which results in lower performance on tasks requiring meta-cognition (Cerqueira et al., 2007); a finding of particular relevance for “at-risk” students (Clark, 2014).

Further, a large sample study among first grade students (*n* = 10, 700) found that students experience the stress of exhausted or anxious teachers vicariously, causing emotional and behavioral problems (Milkie and Warner, 2011). This is a manifestation of “emotional-motor resonance” (Preston and de Waal, 2002); a phenomenon that neuroscientists propose to be a “phylogenetically early system for empathy” (Molnar-Szakacs and Uddin, 2013). The term “resonance” implies a cognitive tension “between” human brains as they seek mutual insights, and jointly monitor social interaction. This shared tension is correlated with coordination between brain activities of inter-actors while they interact (Jackson et al., 2006; Dumas et al., 2010).

One view of stress in the classroom is to view it as a “psychological lesion” (Nelson, 1999, p. 43), which promotes maladaptive learning and diminishes the self-efficacy required to drive meta-cognitive learning strategies. It has already been noted that environmental stress may impede healthy PFC development and functioning. However, it is important to note that stress does not of itself predict low task performance. Indeed, it may enhance learning in supportive contexts (Boyce and Ellis, 2005), underscoring the influence of supportive learning environments. Supportive learning environments by definition engage and excite the learner by emphasizing the idea of equitable social and learning interactions (Clark, 2014; Clark and Dumas, 2015). When human brains experience exciting and novel events they produce stress hormones (e.g., epinephrine) that improve recall (McIntyre and Roozendaal, 2007). The ability to recall information is a basic condition required for the execution of meta-cognitive strategies (Koriat, 2007). The relationship between the learning environment, PFC connectivity and meta-cognition is important because meta-cognitive information processing skills allow learners to acquire and use SRL strategies predictive of successful learning outcomes (Zimmerman and Pons, 1986; Diamond, 2002). In summary, it is in supportive learning environments that the human brain functions in a way most conducive to reflective self-regulation (Blair, 2010).

## Conclusion

Prefrontal cortex plays a pivotal role in the meta-cognitive control over task performance (Anderson et al., 2011; Chein and Schneider, 2012; Fleming and Dolan, 2012; Garrison, 2014). Chein and Schneider (2012) note that the meta-cognitive architecture of the human brain has evolved across the last 150,000 years, expanding by 300–700% over the course of human evolution. It is this that explains the higher domed shape at the front of the human skull in contrast to other large primates. Recent research has provided empirical evidence that the meta-cognitive system: (a) re-configures the brain as it prepares to execute existing routines; and, (b) monitors cognition during the acquisition of new learning strategies. Although there is a lack of clarity about the manner in which different aspects of the PFC interact internally and with other distal substrates (e.g., PPC, Cf. Figure 1) there is consensus that “the region most clearly implicated as a component of the meta-cognitive system is…the anterior pre-frontal cortex” (Chein and Schneider, 2012, p. 82).

“A fundamental goal of education is to equip students with the self-regulatory capabilities that enable them to educate themselves” (Bandura, 1997, p. 174). This is a goal of particular importance in a world characterized by rapid technological change, cultural fragmentation and ecological responsibility. Learners therefore encounter novel situations that entail the recruitment of the meta-cognitive architecture in order to support: (a) the acquisition and integration of new learning strategies; and, (b) the accurate selection and use of strategies. Consequently, empirical studies find high levels of PFC activity during the acquisition of new knowledge, which then recedes to baseline after these new rules have been acquired and integrated (Cole et al., 2010).

The notion that accurate autonoetic (self) knowledge has value, and is something to master has preoccupied thinkers since the time of Socrates. Yet, that quality of self-knowledge required for learners to plan, monitor and reflectively evaluate task performance accurately is often absent. Even in learners with healthy brains, self-assessments are inaccurate (Kruger and Dunning, 1999) and consistently poorer than assessments applied to others leading to what Pronin (2007) termed the “introspection illusion.” A recent study has shown that this illusion may be manipulated to create higher levels of self-efficacy that support task performance in real and measurable terms (Zacharopoulos et al., 2014). This again supports a common call for equitable learning environments in which students feel confident, supported and respected by their peers and school staff (Clark and Dumas, 2015).

### Conflict of interest statement

The authors declare that the research was conducted in the absence of any commercial or financial relationships that could be construed as a potential conflict of interest.
